# Configuration Weights in Coupled-Cluster Theory

**DOI:** 10.1021/acs.jpca.4c07443

**Published:** 2025-03-03

**Authors:** Håkon Emil Kristiansen, Håkon Kvernmoen, Simen Kvaal, Thomas Bondo Pedersen

**Affiliations:** † Hylleraas Centre for Quantum Molecular Sciences, Department of Chemistry, University of Oslo, P.O. Box 1033 Blindern, N-0315 Oslo, Norway; ‡ Department of Physics, University of Oslo, P.O. Box 1033 Blindern, N-0315 Oslo, Norway

## Abstract

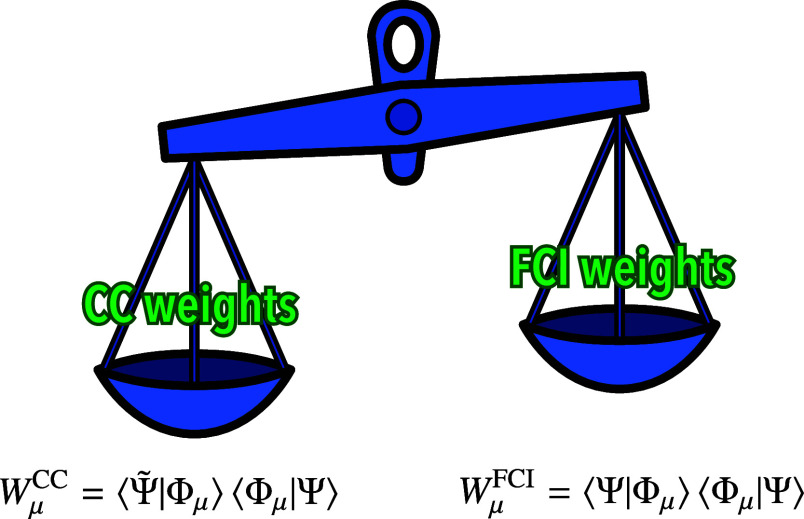

We introduce a simple definition of the weight of any given Slater determinant in the coupled-cluster state, namely as the expectation value of the projection operator onto that determinant. The definition can be applied to any coupled-cluster formulation, including conventional coupled-cluster theory, perturbative coupled-cluster models, nonorthogonal orbital-optimized coupled-cluster theory, and extended coupled-cluster theory, allowing for wave function analyses on par with configuration-interaction-based wave functions. Numerical experiments show that for single-reference systems the coupled-cluster weights are in excellent agreement with those obtained from the full configuration-interaction wave function. Moreover, the well-known insensitivity of the total energy obtained from truncated coupled-cluster models to the choice of orbital basis is clearly exposed by weights computed in the -transformed determinant basis. We demonstrate that the inseparability of the conventional linear parametrization of the bra (left state) for systems composed of noninteracting subsystems may lead to ill-behaved (negative or greater than unity) weights, an issue that can only be fully remedied by switching to extended coupled-cluster theory. The latter is corroborated by results obtained with quadratic coupled-cluster theory, which is shown numerically to yield a significant improvement.

## 1 Introduction

The coupled-cluster (CC) method is arguably the most successful and widely used correlated wave function model in molecular electronic-structure theory, for excited states as well as for ground states^1−7^ and, in recent years, also for many-electron dynamics^8^ induced by external forces such as ultrashort laser pulses. The key to its success is the nonunitary exponential parametrization which—in combination with a nonvariational wave function optimization—yields an inherently size-extensive and size-consistent hierarchy of wave function approximations that converges to the formally exact full configuration-interaction (FCI) theory.

The CC wave function is, however, substantially harder to interpret in elementary quantum-mechanical terms than the FCI wave function. The latter can be written as a superposition
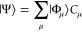
1where the summation is over all N-electron Slater determinants |Φ_μ_⟩ that can be constructed with a given set of orthonormal spin orbitals. The coefficients *C*_μ_ of the ground-state wave function are computed from the variation principle and form the eigenvector corresponding to the lowest eigenvalue of the Hamiltonian matrix in the determinant basis. Evidently, each coefficient *C*_μ_ = ⟨Φ_μ_|Ψ⟩ is the quantum-mechanical probability amplitude for the system being in the N-electron quantum state represented by the Slater determinant |Φ_μ_⟩. The normalization condition
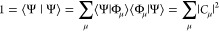
2allows one to judge the relative importance of each determinant |Φ_μ_⟩ in the expansion (1) by its weight (probability) |*C*_μ_|^2^. This gives rise to the commonly used terminology of single-reference (a single dominant determinant or configuration) and multireference (multiple significant configurations) wave functions, typically associated with dynamical and nondynamical electron correlation, respectively. In time-dependent FCI (TDFCI) theory, the coefficients become explicitly time-dependent and can be related to the population of stationary states and interference phenomena during the correlated many-electron dynamics.

It must be kept in mind that the weights are not invariant under rotations of the spin–orbital basis and, therefore, the FCI wave function may appear to be single-reference in one basis but multireference in another one spanning the same Hilbert space. For example, it is well-known that the shortest possible expansion is obtained in the FCI natural-orbital basis.^9^ Recently, since the FCI natural-orbital basis is unknown in practice, orbital localization and other unitary transformations have been proposed to compress the wave function expansion in the context of active-space configuration-interaction theories.^10−13^ Although configuration weights depend on the chosen orbital basis and, therefore, generally cannot be used as a strict diagnostic of single- or multireference character of an electronic state, they are practically the only tools available to us for characterizing wave functions in terms of electronic configurations. It is, therefore, of interest to define CC configuration weights in a manner that converges to the FCI limit while being applicable also to those CC approximations for which a wave function is not strictly defined.

The CC wave function is given by
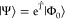
3where the cluster operator
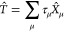
4is defined in terms of amplitudes τ_μ_ and excitation operators . The excitation operators are defined with respect to a chosen reference determinant |Φ_0_⟩ such that  and ⟨Φ_μ_|Φ_ν_⟩ = δ_μν_. The cluster amplitudes τ_μ_ are determined nonvariationally by projection of the Schrödinger equation onto the determinant basis generated by the excitation operators included in the cluster operator. When the cluster operator is truncated, the reference determinant |Φ_0_⟩ should be chosen as the one dominating the (typically unknown) FCI expansion in the same orbital basis. If the reference determinant is not dominant, the truncated CC wave function tend to be a poor approximation unless the single excitations, which effectively act as orbital relaxation parameters, are able to correct a poorly chosen reference. The CC wave function is not normalized but as long as all possible excitations are retained in the cluster operator (4), eqs 1 and 3 are equivalent up to a normalization constant, provided that the reference is not orthogonal to the exact ground-state wave function. The main advantage of the exponential parametrization of CC theory is that it conserves crucial properties of the exact wave function—namely, size consistency and size extensivity^4,7^—when the cluster operator is truncated. These properties are lost when the expansion (1) is truncated, causing dramatic failures that only grow worse as the system size increases.

Unfortunately, there is no simple quantum-mechanical interpretation of the cluster amplitudes. It is, of course, possible to compute the overlap of the CC wave function and any Slater determinant, ⟨Φ_μ_|Ψ⟩, but it cannot be interpreted as a probability amplitude unless the missing normalization is taken into account. This is effectively the same as mapping the CC wave function onto a FCI wave function with the remarkable result that the CC wave function has components in the entire N-electron space regardless of the truncation level of the cluster operator. Even if the cluster operator is truncated, the calculation of CC probability amplitudes by mapping onto the FCI wave function scales factorially with N and is, therefore, (almost) never done in practice.

The fact that the CC wave function has components in the entire N-electron space is commonly used to explain why CC ground-state energies converge faster to the FCI limit than the analogous CI expansions. It should be recalled, however, that the CC energy
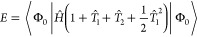
5only has contributions from the reference, single-excited, and double-excited determinants since the Hamiltonian *Ĥ* has excitation rank 2 (i.e., is a two-electron operator). Here, the cluster operator is recast as a sum over excitation ranks from 1 to N
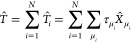
6Equation 5 is valid for any truncation of the cluster operator (including after singles where ) and the triple and higher-order excitations only affect the energy indirectly through the amplitude equations.

Any other observable is computed with the aid of a dual state defined such that the CC expectation-value functional fullfils the Hellman–Feynman theorem. While often perceived as nothing but a computationally convenient construction, the dual state plays an important role for the fundamental physical content of the theory. This is particularly evident in time-dependent CC (TDCC) theory^8^ which is best formulated in the bivariational framework of Arponen,^14^ effectively mapping the quantum-mechanical problem onto classical Hamiltonian mechanics.^15−17^ In this formulation, it is clear that |Ψ⟩ and its dual together form a phase space, indicating that the CC description of a quantum state requires both. This is also evident from equation-of-motion CC (EOM-CC)^18−20^ theory where both left and right eigenstates are needed to compute ground- and excited-state properties and transition probabilities. The relation between TDCC theory and Hamiltonian mechanics was exploited in ref (21) to propose stable symplectic integration of the TDCC equations of motion and to guide physical interpretation of the TDCC quantum state using both |Ψ⟩ and its dual on an equal footing. Moreover, the bivariational viewpoint allows for a simple definition of stationary-state populations as expectation values of suitable projection operators, thus enabling conventional quantum-mechanical interpretations of TDCC quantum dynamics.^22^ Analogously, in the present work, we use the bivariational formulation of CC theory to propose expectation-value expressions for the weights |*C*_μ_|^2^, which are equally valid for truncated cluster operators and at the FCI limit. This allows for a simple interpretation of the CC state on the same footing as configuration-interaction based wave functions.

## 2 Theory

### 2.1 Configuration Weights in Bivariational Theory

Arponen’s bivariation principle^14^ is based on independent appoximations for the wave function, |Ψ⟩, and its hermitian conjugate, denoted , which are canonical variables analogous to the generalized positions and momenta defining the classical phase space,^15−17^ and satisfy the normalization condition . By analogy with the classical phase space, both the ket and the bra are needed to represent the quantum state of the *N*-electron system. In other words, the bra  is as physical as the ket |Ψ⟩ and both must be taken into account in the quantum-mechanical interpretation. It is not sufficient to consider only the ket |Ψ⟩. While this is perhaps an unusual viewpoint for ground-state theories, the EOM-CC^18−20^ approach to excited states operates with “left” (bra) and “right” (ket) eigenstates, both of which are required to compute transition probabilities and ground- and excited-state properties.^19^

Choosing a particular inner product on the CC phase space, the expectation-value function becomes^21^
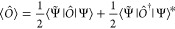
7for some operator *Ô*. Importantly, the bivariation principle guarantees that this expression fullfils both the ordinary time-independent^23,24^ and the time-dependent^25^ Hellmann–Feynman theorem.^14,17,21^

By analogy with eq 2, we define the weight *W*_μ_ of a determinant |Φ_μ_⟩ in the bivariational state as the expectation value of the projection operator 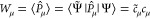
8where we have assumed real orbitals and cluster amplitudes, and introduced
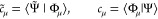
9By the resolution of the identity,  where the summation is over all N-electron Slater determinants in the given spin–orbital basis, we have
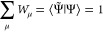
10which suggests that the bivariational weights may be interpreted in the same way as the FCI weights, i.e., as quantum-mechanical probabilities. Note, in particular, that one may compute the weights in a different Slater-determinant basis than that used to compute the wave function. In general, any similarity transformation  can be applied, including unitary orbital rotations, such that
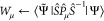
11although doing so may result in intractable computational costs.

One notable caveat arising from the bivariational formulation is that, while inherently real and guaranteed to sum to unity, the individual weights *W*_μ_ are not bounded below by 0 nor above by 1 except at the FCI limit. The unboundedness is a common feature of non-Hermitian theories and is also present in, e.g., EOM-CC theory where transition probabilities may be negative or greater than unity and where closely related sum rules such as the Thomas–Reiche–Kuhn^26−28^ and Condon^29^ sum rules for oscillator strengths and rotatory strengths, respectively, are not fulfilled except at the FCI limit (and with a complete orbital basis).^30−33^ Moreover, we note that the same unboundedness also arises in CC stationary-state populations—but no practical issues were observed in the initial quantum-dynamics studies reported by Pedersen et al.^22^

Of particular interest for comparisons between different methods is the reference weight *W*_0_ and the total weights of singles, doubles, etc., which we define as
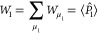
12
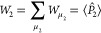
13and so on. Here, we have introduced the total projection operators onto singles, , and onto doubles, . Similar definitions apply for triples (*W*_3_), quadruples (*W*_4_), and higher-order excitations.

Related to the important requirement of size-consistency and size-extensivity, the weights should behave in specific ways when the system is composed of noninteracting subsystems. For simplicity and without loss of generality, we consider an electronic system composed of two infinitely separated (and hence noninteracting) subsystems *A* and *B*. In FCI theory, the wave function is multiplicatively separable, i.e., |Ψ⟩ = |Ψ^*A*^⟩|Ψ^*B*^⟩, and expectation values become either multiplicatively or additively separable when the operator in question is multiplicatively or additively separable, respectively, see, e.g., refs (34 and 35) for very detailed and general discussions of separability (in the context of vibrational CC theory). In bivariational theory, ideally, the bra  should be multiplicatively separable, too. Now, the determinant projection operators are not generally separable, neither multiplicatively nor additively, since an excitation may be either localized on subsystem *A* or on subsystem *B*, or involve spin orbitals on both subsystems. Assuming that the chosen reference determinant is multiplicatively separable, we have the relations
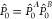
14
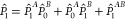
15
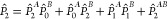
16for the projection operators onto the reference, singles, and doubles, respectively. Hence, as long as both  and |Ψ⟩ are multiplicatively separable (as in FCI theory), the corresponding weights can be expressed in terms of subsystem weights according to
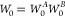
17
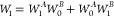
18
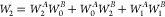
19These relations make it abundantly clear that weights are not size-extensive quantities and, hence, cannot be used as a rigorous diagnostic for single- or multireference character. At the very least, one would have to use the ratio of the two largest weights, although this measure remains orbital-dependent. On the other hand, if one observes a reference weight close to unity in a given spin–orbital basis, then the system certainly can be characterized as single-reference. For the He atom, for example, with the aug-cc-pVDZ basis set and canonical HF spin orbitals, the FCI reference weight is *W*_0_ = 0.992, leaving no doubt that the electronic wave function is single-reference. However, the wave function would still be single-reference for 860 noninteracting He atoms even though the reference weight would drop to *W*_0_ = 0.001. More general approaches to the characterization and error assessment of the specific case of CC wave functions have been developed recently. Bartlett et al.^36^ introduced size-extensive and orbital-invariant multideterminant and multireference indices for characterizing CC wave functions, and Faulstich et al.^37^ proposed a diagnostic based on mathematical analysis of CC theory. Still, despite its weaknesses, the weight concept plays a fundamental role in the understanding of electronic structure and, for example, the dominant weights are commonly used to describe the wave function obtained from a complete active space self-consistent field calculation in a given orbital basis.

In the following sections we will discuss weights in the context of various flavors of CC theory.

### 2.2 Conventional CC Theory

The most widely employed CC formulation in quantum chemistry uses the parametrization of eq 3 with the reference determinant typically chosen to be the ground-state HF determinant, and
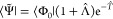
20Here, the de-excitation cluster operator is defined in terms of amplitudes λ_μ_ as
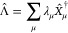
21where the summation is the same as in the cluster operator, eq 4. Systematic truncation of the cluster operators lead to a hierarchy of increasingly accurate models. For example, the CC singles (CCS), CC singles and doubles (CCSD), CC singles doubles and triples (CCSDT) models are obtained by truncating the cluster operators after singles (, ), after doubles (, ), and after triples (, ), respectively.

The bivariation principle requires that the CC Lagrangian (i.e., energy functional)
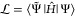
22be stationary with respect to variations in the amplitudes λ and τ. This leads to the equations
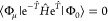
23
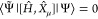
24which determine the τ and λ amplitudes. Note that eqs 23 and 24 are uncoupled such that the λ amplitudes can be regarded as functions of the cluster amplitudes τ and of the Hamiltonian *Ĥ*. This is a direct consequence of the linear parametrization of Λ̂ in eq 21. The λ amplitudes can be viewed as Lagrange multipliers that ensure stationarity of the CC energy  under the constraints defined by eq 23.^2,38−40^ The Lagrangian point of view has been demonstrated to yield significant computational advantages through the so-called 2*n* + 1 and 2*n* + 2 rules, which show that the τ amplitudes to order *n* in perturbation theory determine the energy through order 2*n* + 1 while the λ amplitudes to order *n* determine the energy through order 2*n* + 2.^2,39,40^ Recently, the Lagrangian technique has been generalized to other properties than the energy, leading to increased accuracy at significantly reduced computational cost.^41^

Alternatively, but equivalently, the linear parametrization of Λ̂ can be viewed as a computationally convenient linear reparameterization of a de-excitation operator involving the resolvent of the similarity transformed Hamiltonian, , arising from the derivative of eq 23 with respect to a perturbation. For more details on this formulation, see ref (7) and references therein.

The linear parametrization of Λ̂ yields a bra, , with obvious similarity to configuration-interaction wave functions. This is unproblematic at the FCI limit (when all excitations are included) but any truncation breaks multiplicative separability of . While expectation values of additively separable operators remain additively separable, those of multiplicatively separable operators are not multiplicatively separable.^34,35^ Since the determinant projection operators are not additively separable (only the reference projector is multiplicatively separable), the linear parametrization of Λ̂ implies that truncated CC weights do not obey eqs 17–19.

Using the definitions (9), we may recast |Ψ⟩ and  as the configuration-interaction expansions
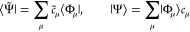
25While the summation in the ket expansion always runs over all N-electron Slater determinants, the summation in the bra expansion ends at the truncation level of Λ̂. Thus, as a direct consequence of the linear de-excitation operator, CC weights are only nonzero up to the truncation level of the cluster operators. For example, for the CCSD model, we have *W*_*n*_ = 0 for *n* > 2, and *W*_0_ + *W*_1_ + *W*_2_ = 1.

The projection operators have excitation rank 0 in a given spin–orbital basis, prohibiting couplings between the components of  and higher-order components of |Ψ⟩. These do play a role in bivariational CC theory, however. Expectation values of Hermitian operators with nonzero excitation rank can be written as
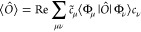
26Within CCSD theory, for example, if *Ô* is a one-electron operator the doubles components of  couple to the triples components of |Ψ⟩. Similarly, for two-electron operators the quadruples components of |Ψ⟩ contribute.

For the CCS model, doubles and higher-order weights vanish and only the reference and singles weights may be nonzero
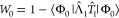
27
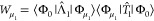
28Note that if the reference determinant is the HF ground-state wave function, the singles amplitudes vanish. For the CCSD model, we obtain
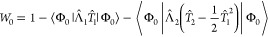
29
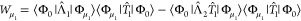
30
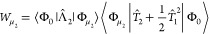
31while the CCSDT weights are given by
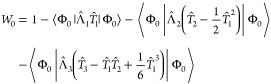
32
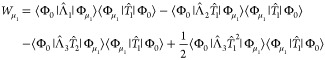
33
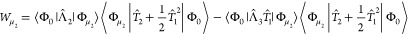
34
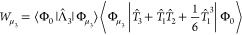
35Detailed expressions in spin–orbital basis are provided in the Appendix.

As is well-known, the single-excitation part of the cluster operator, , acts as an approximate orbital-relaxation operator, making the CC ground-state energies relatively insensitive to the choice of spin–orbital basis.^4,7,42^ At the FCI limit, the CC method becomes fully orbital invariant provided that the chosen reference determinant is not orthogonal to the FCI wave function. The effect of single excitations can be elucidated by weights obtained from similarity-transformed projection operators using eq 11 with . The singles weights vanish identically in this projection basis, whereas the reference weight is expected to increase compared with the untransformed basis.

### 2.3 Alternative Formulations

Since the bivariation principle is based on independent approximations for the bra and ket functions, one might apply alternative expectation-value functionals based on either  or |Ψ⟩ alone, i.e.
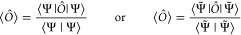
36Results computed from either of these expressions will be identical to those computed from eq 7 at the FCI limit. With truncated cluster operators, however, the different expectation-value functionals will produce different results. For the Hamiltonian, for example, the expectation-value functional should reproduce the CC energy *E*. However
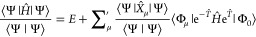
37
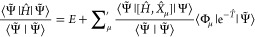
38
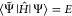
39where primes indicate summations over excitations not included in the cluster operator (e.g., triples and higher-order excitations for the CCSD model), and where we have assumed that  and |Ψ⟩ are real-valued functions. At the FCI limit, it follows from eqs 23 and 24 that all three expressions yield *E* but only the bivariational expectation-value functional reproduces the correct energy with truncated cluster operators.

For configuration weights, the three expectation-value expressions are identical to leading (i.e., second) order in the amplitudes if one assumes 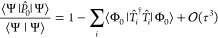
40
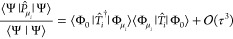
41
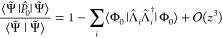
42
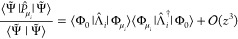
43
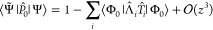
44
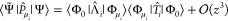
45where *z* denotes λ and τ amplitudes collectively, and the summations are over the excitation ranks included in the cluster operators. Thus, to leading order in the amplitudes, configuration weights above the truncation level of the cluster operators vanish with either of the three expressions.

The |Ψ⟩ and  expectation-value functionals yield weights that are bounded below by 0 and above by 1 regardless of the truncation level of the cluster operators. The former can be computed from τ amplitudes alone, while the latter also requires the λ amplitudes. The weights obtained from |Ψ⟩ are generally nonzero in the entire *N*-body Hilbert space, whereas the  weights are nonzero only for excitations within the truncation level of the cluster operators. Thus, computing weights from |Ψ⟩ alone has FCI complexity regardless of the truncation, necessitating approximations such as, e.g., truncating the linear re-expansion of |Ψ⟩ at some chosen excitation level. This is unfortunate since the full |Ψ⟩ expectation-value functional is required to ensure the correct separability properties regardless of the cluster-operator truncation.

More importantly, only the bivariational expectation-value functional is in agreement with the Hellmann–Feynman theorem at any truncation level. This makes it preferable over the other two expressions for the calculation of ground-state properties in CC theory, including configuration weights. As we shall see below, this choice also allows us to define configuration weights for perturbation theories where a wave function is not explicitly defined. Finally, as discussed by Stanton and Bartlett,^19^ we stress that the bivariational expectation-value functional emerges naturally from EOM-CC theory and thus allows us to define configuration weights for excited states as well as the ground state within a single common framework.

### 2.4 CC Perturbation Theories

Some of the most widely used CC methods are based on perturbation theory and, as such, do not involve an explicit wave function parametrization. Examples include the popular second-order Møller–Plesset (MP2)^2,43^ theory and the related second-order approximation to CCSD theory, the CC2 model,^44^ and the fourth-order approximation to full triples treatment, the CC3 model,^45^ which is often considered to be of benchmark quality, especially for response properties and excitation energies.^46^ Also the “Gold Standard” method of quantum chemistry, the CCSD method with perturbative connected triples correction (CCSD(T)),^47^ belongs to the set of approximations that do not provide an explicit wave function expression.

Even in the absence of explicit wave function expressions, one can still use the expectation-value approach. One simply starts from the bivariational energy functional and defines the expectation-value functional in agreement with the Hellman–Feynman theorem. Replacing the Hamiltonian operators with projection operators then leads to configuration weights for such perturbative CC methods.

For the CC2 model, the energy functional is given by^44^
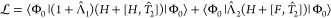
46where we have introduced the notation
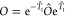
47for -transformed operators, *F̂* is the Fock operator, and |Φ_0_⟩ is the canonical HF ground-state determinant. Replacing *Ĥ* and *F̂* with projection operators, we obtain the same expressions for the reference, singles, and doubles weights as for the CCSD model above, eqs 29–31. The only difference is that the amplitudes are evaluated from the CC2 equations rather than the CCSD ones.

Similarly, the CC3 energy functional is defined as^45^
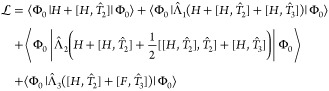
48from which one easily obtains weights by replacing *Ĥ* and *F̂* with projection operators. The resulting expressions for the weights differ from the CCSDT ones in eqs 32–35 and are given by
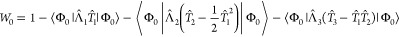
49
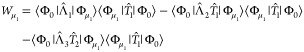
50
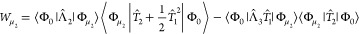
51
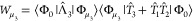
52Compared with CCSDT, the missing terms in the CC3 weights are those that involve  in conjunction with higher-order singles which are removed in the perturbation expansion defining the CC3 model.

The CC2 and CC3 models are mainly aimed at time- or frequency-dependent properties such as dynamic polarizabilities and hyperpolarizabilities. For ground-state energies, they usually can be replaced by noniterative perturbation theories such as MP2 and CCSD(T), respectively. For the MP2 model, the energy functional is defined by
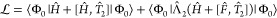
53leading to the weights
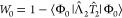
54
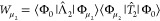
55The singles weights vanish (*W*_μ__1_ = 0) and  in MP2 theory. The CCSD(T) energy functional can be written as^48^
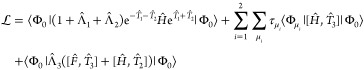
56from which we obtain
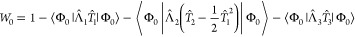
57
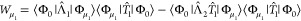
58
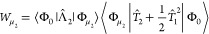
59
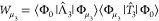
60The expressions for the MP2 weights are identical to those obtained in CCD theory (the CCSD expressions with ). The CCSD(T) weight expressions, on the other hand, differ from both CC3 and full CCSDT by the lack of all disconnected triples contributions. Since the CCSD(T) model consists of an energy correction from perturbative connected triples only, the expressions for the singles and doubles weights are identical to those obtained from CCSD theory. The computed singles and doubles weights are different, however, since the λ_μ_1__ and λ_μ_2__ amplitudes are affected by the perturbative triples corrections in eq 56.

### 2.5 Nonorthogonal Orbital-Optimized CC Theory

Orbital relaxation can be included explicitly in the CC formulation by replacing  with an orbital-rotation operator , where
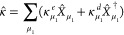
61The bivariational CC Ansatz then becomes
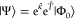
62
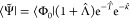
63where singles are excluded from the cluster operators *T̂* and Λ̂. By restricting κ̂ to be anti-Hermitian, , such that the orbital-rotation operator is unitary, we obtain the orbital-optimized CC (OCC) model.^32,42,49−51^ The OCC model, however, fails to converge to the FCI limit for systems with more than two electrons.^52^ As demonstrated by Myhre,^53^ this issue can be removed by lifting the anti-Hermiticity restriction on κ̂, yielding the nonorthogonal orbital-optimized CC (NOCC) theory^54,55^ (or its active-space generalization coined orbital-adaptive time-dependent CC (OATDCC)^56^ theory).

The NOCC equations are identical to the conventional CC eqs 23 and 24 with singles amplitudes removed and with the Hamiltonian replaced by the similarity-transformed operator , while the orbital-rotation parameters κ^*e*^ and κ^*d*^ are determined by generalized Brillouin conditions.^54^ The four sets of equations are coupled and must be solved simultaneously—i.e., the λ amplitudes are no longer given as functions of the τ amplitudes and, hence, cannot be viewed as Lagrangian multipliers.

The NOCC configuration weights can easily be computed from eq 11 with , which implies that singles weights are identically zero. Projection onto the untransformed Slater determinants—typically chosen to be HF determinants—is not generally feasible, as it would require a computational effort comparable to a FCI calculation. Truncating the cluster operators after double excitations gives the NOCC doubles (NOCCD) model for which weights can be computed using the CCSD expressions in eqs 29 and 31 with  (see the Appendix for full detail). Note that weights beyond doubles vanish in NOCCD theory since the de-excitation cluster operator Λ̂ remains linear in truncated NOCC theory. By the same token,  is not multiplicatively separable in truncated NOCC theory and, hence, the NOCC weights do not obey eqs 17 and 19 for noninteracting subsystems.

### 2.6 Quadratic CC Theory

The only generally applicable way to ensure separability of  is to replace the linear de-excitation cluster operator with an exponential operator
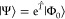
64
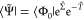
65where , including singles in both *T̂* and Σ̂. This Ansatz defines extended CC (ECC) theory, which was proposed and analyzed in detail by Arponen and co-workers.^14,57,58^ The ECC equations are significantly more complicated and computationally demanding than the conventional CC equations and, therefore, applications have been scarce.^59−65^ Multiplicative separability at any truncation level of  as well |Ψ⟩ was explicitly demonstrated by Hansen et al.^35^ Their work aimed at vibrational ECC theory but applies to electronic systems as well. Hence, the weights in truncated (as well as untruncated) ECC theory behave correctly for noninteracting subsystems.

Rather than the full ECC method, we will in the present work consider the quadratic CC (QCC)^66,67^ method obtained by expanding the exponential de-excitation operator in eq 65 to second order, i.e.
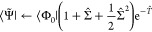
66Truncation after doubles yields the QCC singles and doubles (QCCSD) model, which includes up to quadruple de-excitations through the quadratic term in eq 66. Hence, up to quadruple-excitation weights are nonzero in QCCSD theory
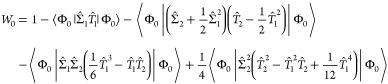
67
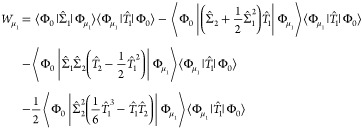
68
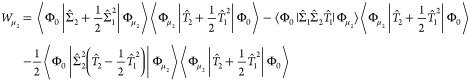
69
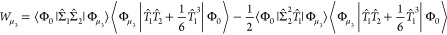
70
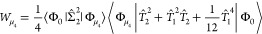
71Detailed expressions for the reference, singles, doubles, triples, and quadruples weights in spin–orbital basis can be found in ref (68) along with the working equations for determining the τ and σ amplitudes.

The truncation of the exponential in eq 66 implies that  is not multiplicatively separable. Nevertheless, the inclusion of the quadratic term is expected to reduce the deviation from separability compared with conventional CCSD theory, especially for four-electron systems where quadruple de-excitations will be important.

## 3 Results

### 3.1 Computational Details

Calculations were performed with the PySCF^69^ and HyQD^70^ program packages using closed-shell spin-restricted implementations of HF and Kohn–Sham (KS) density-functional theory. For the latter, we used the Tao–Perdew–Staroverov–Scuseria hybrid density functional (TPSS0)^71,72^ with 25% HF exchange, as implemented in the libxc software library.^73^ All electrons were correlated unless stated otherwise. Both the correlation-consistent double- and triple-ζ basis sets cc-pVDZ and cc-pVTZ were used.^74,75^ In a few cases, we also used the 6-31G basis set.^76^ All basis set definitions are taken from the Basis Set Exchange.^77−79^

### 3.2 Validation of the CC Weight Concept

We start by comparing the weights obtained from the conventional CCSD method with those obtained from FCI theory, using the restricted HF (RHF) reference determinant in both cases. Table 1 lists the reference, singles, and doubles weights for the atoms He, Be, Ne, and Ar obtained with the CCSD method and their difference with respect to the FCI results, Δ*W*_*n*_ = *W*_*n*_^CCSD^ – *W*_*n*_^FCI^, along with the energy difference, Δ*E* = *E*^CCSD^ – *E*^FCI^. As expected, the CCSD and FCI results are identical (to within convergence thresholds) for the He atom, which is evidently a single-reference problem with a reference weight of 99.2%, essentially no singles weight, and 0.8% doubles weight. Also the Ne and Ar atoms are clear-cut single-reference problems, with reference weight above 95% and less than 5% doubles weight, in excellent agreement with FCI theory where higher-order excited determinants are negligible.

**Table 1:** CCSD Reference, Singles, and Doubles Weights for Selected Closed-Shell Atoms and Errors Relative to FCI Results^a^

atom	basis set	Δ*E*	*W*_0_^CCSD^	Δ*W*_0_	*W*_1_^CCSD^	Δ*W*_1_	*W*_2_^CCSD^	Δ*W*_2_
He	cc-pVTZ	0.0	0.99216	0.00000	0.00001	0.00000	0.00784	0.00000
Be	cc-pVTZ	0.3	0.90817	0.00096	0.00143	0.00000	0.09040	–0.00090
Ne	cc-pVDZ	1.2	0.97256	0.00022	0.00004	0.00000	0.02740	0.00026
Ar	cc-pVDZ	1.5	0.95149	0.00047	0.00001	0.00000	0.04850	0.00067

a The Ne core of the Ar atom is kept frozen in the correlation treatment. Energy differences are given in mE_h_.

The agreement with FCI theory is only slightly worse for the Be atom, which has about 9% doubles weight and 91% reference weight. The CCSD method predicts that two doubly excited configurations contribute significantly to *W*_2_ in this case, |1s^2^2p^2^⟩ with weight 0.044 (49.10% of *W*_2_) and |1s^2^2p3p⟩ with weight 0.035 (38.99% of *W*_2_), in good agreement with the FCI weights 0.045 (49.11% of *W*_2_) and 0.036 (39.04% of *W*_2_), respectively. Using the restricted KS (RKS) orbital basis instead of the RHF one leads to a CCSD energy decrease by just 2.7 μE_h_ (7.1 J/mol). The reference, singles, and doubles weights are virtually unchanged but the distribution of doubles weight between the |1s^2^2p^2^⟩ and |1s^2^2p3p⟩ configurations is changed to 77% and 16%, respectively.

More validation data can be found in Tables 2–5 for diatomic molecules at different internuclear distances.

**Table 2:** Reference, Singles, and Doubles Weights for the H_2_ Molecule Obtained With the cc-pVTZ Basis Set^a^

	*R*/*R*e	MP2	CC2	CCSD	FCI
*W*_0_	1	0.98987	0.98975	0.98209	0.98209
	3	0.92553	0.90779	0.71195	0.71195
	6	0.30763	0.09465	0.48444	0.48444
*W*_1_	1	0.00000	0.00009	0.00012	0.00012
	3	0.00000	0.00715	0.01474	0.01474
	6	0.00000	0.04904	0.02355	0.02355
*W*_2_	1	0.01013	0.01015	0.01779	0.01779
	3	0.07447	0.08506	0.27331	0.27331
	6	0.69237	0.85631	0.49201	0.49201
Δ*E*	1	7.695	7.601	0.000	
	3	53.562	49.204	0.000	
	6	28.153	–1.073	0.000	

a The equilibrium bond distance is *R*_e_ = 1.4 a_0_. Energy differences are reported in mE_h_ and the FCI energies are −1.17233459 E_h_ at *R*_e_, −1.01096374 E_h_ at 3*R*_e_, and −0.99963751 E_h_ at 6*R*_e_.

Table 2 shows that the CCSD and FCI weights agree for the H_2_ molecule, also at stretched bond lengths, as they should for a two-electron system. At 6*R*_e_, the doubles weight is dominated by the |σ_u_^2^⟩ configuration and is roughly equal to the |σ_g_^2^⟩ reference weight, as expected. The MP2 and CC2 weights are excellent approximations at *R*_e_ but quickly deteriorate as the bond length is increased. This is caused by the diminishing gap between the occupied σ_*g*_ orbital and the virtual σ_*u*_ orbital at stretched bond lengths, causing overstimation of the dominant doubles amplitudes by the second-order perturbation treatment. This is also reflected in the energy errors, which initially increase with *R* and subsequently decrease such that the energy eventually falls below the FCI one. This is an archetypical failure of perturbation theory.

For the LiH molecule the CCSD and FCI energies and weights are in very good agreement, see Table 3. The reference weight, corresponding to the configuration |1σ^2^2σ^2^⟩, is 0.969 at the equilibrium distance *R*_e_, decaying to 0.823 and 0.397 at 2*R*_e_ and 3*R*_e_, respectively. At the stretched geometries, there are significant contributions from both singles and doubles, while the triples and quadruples weights remain small (<0.001) and essentially negligible. The singles weight mainly comes from the configuration |1σ^2^2σ3σ⟩ with a weight of 0.046 at 2*R*_e_ and 0.280 at 3*R*_e_ in the FCI wave function. The corresponding CCSD singles weight is 0.045 at 2*R*_e_ and 0.277 at 3*R*_e_. In the FCI wave function, the dominating double-excited configurations are |1σ^2^3σ^2^⟩ and |1σ^2^3σ4σ⟩ with weights of 0.017 and 0.027 at 2*R*_e_, and 0.162 and 0.086 at 3*R*_e_ in the FCI wave function. The corresponding CCSD doubles weights are 0.016 and 0.027 at 2*R*_e_, and 0.161 and 0.085 at 3*R*_e_.

**Table 3:** Reference, Singles, Doubles, Triples, and Quadruples Weights for the LiH Molecule Obtained with the cc-pVTZ Basis Set^a^

*R*/*R*_e_	MP2	CC2	CCSD	CCSD(T)	CC3	CCSDT	FCI
*W*_0_							
1	0.98383	0.98359	0.96855	0.96840	0.96841	0.96837	0.96842
2	0.96921	0.94258	0.82731	0.82316	0.82498	0.82440	0.82456
3	0.91068	0.62084	0.39707	0.33539	0.39154	0.39024	0.39100
*W*_1_							
1	0.00000	0.00015	0.00040	0.00041	0.00041	0.00041	0.00041
2	0.00000	0.01732	0.05577	0.05806	0.05692	0.05720	0.05719
3	0.00000	0.19444	0.29819	0.34268	0.30134	0.30207	0.30174
*W*_2_							
1	0.01617	0.01626	0.03105	0.04317	0.03117	0.03120	0.03110
2	0.03079	0.04010	0.11691	0.11874	0.11801	0.11829	0.11794
3	0.08932	0.18472	0.30474	0.32155	0.30690	0.30740	0.30623
*W*_3_							
1	0.00000	0.00000	0.00000	0.00001	0.00001	0.00002	0.00002
2	0.00000	0.00000	0.00000	0.00004	0.00009	0.00011	0.00013
3	0.00000	0.00000	0.00000	0.00038	0.00023	0.00029	0.00057
*W*_4_							
1	0.00000	0.00000	0.00000	0.00000	0.00000	0.00000	0.00005
2	0.00000	0.00000	0.00000	0.00000	0.00000	0.00000	0.00017
3	0.00000	0.00000	0.00000	0.00000	0.00000	0.00000	0.00045
Δ*E*							
1	10.719	10.590	0.082	0.014	0.015	0.0003	
2	20.333	18.484	0.201	0.006	0.043	0.004	
3	46.820	25.834	0.644	–1.196	0.123	0.008	

a The equilibrium bond distance is *R*_e_ = 3.037 a_0_. Energy differences are reported in mE_h_ and the FCI energies are −8.03664666 E_h_ at *R*_e_, −7.96676083 E_h_ at 2*R*_e_, and −7.94676936 E_h_ at 3*R*_e_.

The MP2 and CC2 approximations overestimate the reference weight with a concomitant underestimation of the doubles weight. This is also reflected in the energy errors which are 2 orders of magnitude greater than the CCSD ones. The CC3 method performs somewhat better than the CCSD(T) approximation, with results closer to the CCSDT and FCI ones. In particular, the CCSD(T) energy falls below the FCI one at 3*R*_e_ while the CC3 energy remains above. The CCSDT energies agree with the FCI ones to within a few μE_h_ at all distances. While triples weights are insignificant, the triples amplitudes clearly influence the reference, singles, and doubles weights, improving the already good agreement with FCI weights at the CCSD level.

Somewhat larger deviations are observed for the HF molecule in Table 4, especially at stretched geometries. The RHF refence configuration |1σ^2^2σ^2^1π^4^3σ^2^⟩ dominates with a weight slightly below 96% at the equilibrium distance. At the stretched geometries, the FCI and CCSD methods agree that two excited configurations—the single-excited |1σ^2^2σ^2^1π^4^3σ4σ⟩ and the double-excited |1σ^2^2σ^2^1π^4^4σ^2^⟩—contribute significantly. Their weights at 2*R*_e_ are 0.026 and 0.131 in the FCI wave function, while the CCSD method predicts 0.021 and 0.110. At 2.5*R*_e_, the FCI and CCSD weights are 0.068, 0.267 and 0.059, 0.233, respectively. Also for the HF molecule, the quality of the second-order approximations decrease as the bond is stretched. The CC3 method performs better than the CCSD(T) approximation, especially at stretched geometries. Although the triples and quadruples weights are small (<0.005), the inclusion of triples in the cluster operators improves the reference, singles, and doubles weights. Overall, therefore, these preliminary investigations indicate a hierarchy of weight approximations following the order MP2 < CC2 < CCSD < CCSD(T) < CC3 < CCSDT. The apparent superiority of the CC3 method over the CCSD(T) approximation is not too surprising, of course, since the latter is aimed at a perturbative correction of the energy while the former is a similar correction of the wave function.

**Table 4:** Reference, Singles, Doubles, Triples, and Quadruples Weights for the HF Molecule Obtained with the cc-pVDZ Basis Set^a^

*R*/*R*_e_	MP2	CC2	CCSD	CCSD(T)	CC3	CCSDT	FCI
*W*_0_							
1.0	0.96013	0.95912	0.95755	0.95620	0.95606	0.95607	0.95654
2.0	0.90859	0.88232	0.82333	0.77994	0.79469	0.79313	0.79455
2.5	0.84137	0.77753	0.66624	0.51708	0.60795	0.61105	0.61869
*W*_1_							
1.0	0.00000	0.00048	0.00038	0.00036	0.00040	0.00041	0.00040
2.0	0.00000	0.01260	0.02499	0.03443	0.02981	0.02992	0.03021
2.5	0.00000	0.02923	0.06599	0.10909	0.08013	0.07704	0.07593
*W*_2_							
1.0	0.03987	0.04040	0.04207	0.04317	0.04327	0.04324	0.04196
2.0	0.09141	0.10507	0.15168	0.18375	0.17358	0.17471	0.16928
2.5	0.15863	0.19324	0.26776	0.36897	0.30813	0.30761	0.29451
*W*_3_							
1.0	0.00000	0.00000	0.00000	0.00027	0.00027	0.00029	0.00027
2.0	0.00000	0.00000	0.00000	0.00187	0.00192	0.00224	0.00235
2.5	0.00000	0.00000	0.00000	0.00486	0.00379	0.00430	0.00492
*W*_4_							
1.0	0.00000	0.00000	0.00000	0.00000	0.00000	0.00000	0.00058
2.0	0.00000	0.00000	0.00000	0.00000	0.00000	0.00000	0.00285
2.5	0.00000	0.00000	0.00000	0.00000	0.00000	0.00000	0.00480
Δ*E*							
1.0	7.391	6.644	2.432	0.491	0.402	0.407	
2.0	27.398	19.085	10.329	0.321	1.611	1.214	
2.5	46.719	28.342	17.444	–6.566	1.957	1.349	

a The equilibrium bond distance is *R*_e_ = 1.737 a_0_. Energy differences are reported in mE_h_ and the FCI energies are −100.23059429 E_h_ at *R*_e_, −100.06493232 E_h_ at 2*R*_e_, and −100.03732519 E_h_ at 2.5*R*_e_.

It is well-known that the CCSD method works well for the systems considered above, at least in terms of the energy. Our investigation shows that the CCSD weights also are good approximations to the FCI weights for these systems. To challenge the CC weight concept, we now turn our attention to the N_2_ molecule, which is single-reference dominated at the equilibrium bond distance and rapidly develops increasing multireference character as the bond is stretched. This should be clearly reflected in the CCSD weights deviating substantially from FCI results as the bond is stretched. Indeed, this is what we observe from the data presented in Table 5 where we have also included results obtained with the CC2, QCCSD, CCSD(T), CC3, and CCSDT models for comparison.

**Table 5:** Reference, Singles, Doubles, Triples, and Quadruples Weights for the N_2_ Molecule Obtained with the 6-31G Basis Set^a^

*R*/*R*_e_	CC2	CCSD	QCCSD	CCSD(T)	CC3	CCSDT	FCI
*W*_0_							
1.0	0.88888	0.89993	0.90053	0.89100	0.89036	0.89107	0.89218
1.3	0.70313	0.79704	0.80077	0.76451	0.76658	0.77094	0.78069
1.6	0.25675	0.33220	0.57363	0.09715	0.28670	0.21930	0.53554
*W*_1_							
1.0	0.00386	0.00217	0.00173	0.00179	0.00202	0.00205	0.00205
1.3	0.01866	0.00574	0.00413	0.00435	0.00562	0.00578	0.00562
1.6	0.05806	0.01245	0.00697	0.01054	0.00862	0.00872	0.00856
*W*_2_							
1.0	0.10727	0.09790	0.09358	0.10516	0.10544	0.10461	0.09852
1.3	0.27820	0.19722	0.17828	0.22478	0.22090	0.21782	0.18699
1.6	0.68519	0.65536	0.33049	0.87494	0.68799	0.75514	0.36339
*W*_3_							
1.0	0.00000	0.00000	0.00013	0.00205	0.00219	0.00227	0.00202
1.3	0.00000	0.00000	0.00068	0.00637	0.00690	0.00547	0.00447
1.6	0.00000	0.00000	0.00363	0.01737	0.01669	0.01684	0.00922
*W*_4_							
1.0	0.00000	0.00000	0.00403	0.00000	0.00000	0.00000	0.00495
1.3	0.00000	0.00000	0.01614	0.00000	0.00000	0.00000	0.02025
1.6	0.00000	0.00000	0.08529	0.00000	0.00000	0.00000	0.07392
Δ*E*							
1.0	–7.206	9.860	7.858	1.925	1.459	2.021	
1.3	–67.969	24.939	18.441	4.891	2.926	7.283	
1.6	–199.877	36.411	29.944	–10.146	–4.053	2.765	

a The equilibrium bond distance is *R*_e_ = 2.102 a_0_. Energy differences are reported in mE_h_ and the FCI energies are −109.10719404 E_h_ at *R*_e_, −109.00405250 E_h_ at 1.3*R*_e_, and −108.89416902 E_h_ at 1.6*R*_e_.

We first note that the FCI weights up to quadruples sum to 0.99972 at *R*_e_, 0.99802 at 1.3*R*_e_, and 0.99063 at 1.6*R*_e_ and, thus, excited determinants beyond quadruples contribute less than 1% at all three distances. The RHF reference determinant is |1π^4^5σ^2^⟩ where, for convenience, we have included only the highest-lying occupied orbitals in the notation. It has a weight of 0.892 in the FCI wave function at the equilibrium distance, dropping rapidly to 0.781 and 0.536 at 1.3*R*_*e*_ and 1.6*R*_e_, respectively. The dominant double-excited configuration in the FCI wave function is |1π^2^5σ^2^2π^2^⟩ with a weight of 0.025 (25% of *W*_2_) at the equilibrium distance, increasing to 0.042 (22%) at 1.3*R*_e_ and 0.173 (48%) at 1.6*R*_e_. Also, at 1.6*R*_e_ the quadruple-excited configuration |5σ^2^2π^4^⟩ becomes non-negligible with a weight of 0.027 (36% of *W*_4_) in the FCI wave function. At *R*_e_ and 1.3*R*_e_ the CCSD and QCCSD weights are in reasonably good agreement with the FCI weights. At 1.6*R*_e_, however, the CCSD method severely underestimates the FCI reference weight and overestimates the doubles weight, indicating a failure of the CCSD method despite an energy error on the same order of magnitude as at the shorter bond distances. On the other hand, the QCCSD model only modifies the bra state compared with the CCSD model and provides a much-improved approximation of the FCI weights with roughly the same energy errors. This can be attributed to the fact that disconnected triples and quadruples are included in . These contribute not only to the triples and quadruples weights but also to the reference, singles, and doubles weights. In addition, the singles and doubles amplitudes are indirectly affected by the quadratic term of the bra through the amplitude equations.

The CC2 weights are quite similar to the CCSD ones, deviating significantly from the FCI ones as the N_2_ bond is elongated, but with much greater energy errors, all below the FCI energy. Including triples in the description improves the energy but does not improve the agreement for the weights. In fact, the reference and doubles weights are even further from the FCI ones than the CCSD weights, especially for the CCSD(T) model, although the CCSD(T), CC3, and CCSDT energies agree to within ∼10 mE_h_ at all three distances.

### 3.3 Effect of Orbital Choice

**Figure 1:**
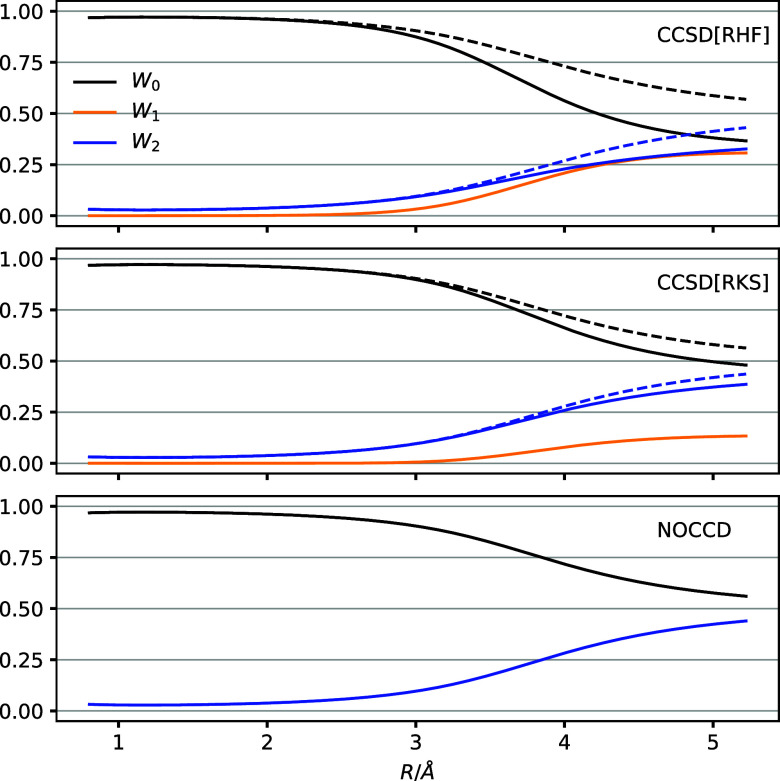
*W*_0_, *W*_1_, and *W*_2_ for the LiH molecule as functions of the bond distance. The top two panels show results obtained from CCSD theory using the RHF and RKS reference determinants; full lines: bare determinant basis, dashed lines: -transformed basis. The last panel shows results obtained in the fully rotated determinant basis with NOCCD theory. The cc-pVTZ basis set is used in all calculations, and smooth curves are obtained by cubic spline interpolation.

Unlike FCI theory, truncated CC models rely on a reference determinant that is close enough to the FCI wave function. It is well-known, however, that the CCSD model can compensate for a poor choice of reference determinant through the approximate orbital relaxation provided by the  operator. This makes the CCSD model (and higher-order truncated CC models) near-invariant to the choice of reference determinant, i.e., to the choice of orbital basis. One typically chooses the HF determinant which may be a poor choice at, e.g., stretched bond lengths. In cases where the HF solution shows pathological behavior, one may try other choices such as the KS determinant and rely on  to approximately rotate the reference determinant into a closer-to-optimal one. The NOCCD model includes a complete biorthonormal orbital rotation and, in essence, thus defines a new reference determinant to which the CCD approach is applied. It should be stressed, of course, that the new reference determinant of the NOCCD model is determined in concert with the correlation. These effects can be illustrated by the CC weight concept. We first consider the LiH molecule for which the CCSD model provides an excellent approximation of the FCI energy across the ground-state potential-energy curve, despite a significant reduction of the reference weight at stretched bond lengths. Figure 1 shows *W*_0_, *W*_1_, and *W*_2_ obtained with the CCSD model using either the RHF reference (denoted CCSD[RHF]) or the KS reference (denoted CCSD[RKS]) with the TPSS0 density-functional approximation. For these methods, the weights are computed by projection onto the bare determinants using  and by projection onto the -transformed determinants using . Finally, we also show the reference and doubles weights obtained from NOCCD theory by projection onto the rotated determinants using .

The potential-energy curves obtained from the CCSD[RHF], CCSD[RKS], and NOCCD models are nearly identical, indicating the approximate orbital invariance. At the equilibrium bond distance, *R*_e_ = 1.596 Å, the CCSD[RKS] and NOCCD energies are 1.04 μE_h_ (2.74 J/mol) and 41.5 μE_h_ (109 J/mol) above the CCSD[RHF] energy. The maximum deviation across the potential-energy curves is 44.2 μE_h_ (116 J/mol) for the CCSD[RKS] method and 210 μE_h_ (552 J/mol) for the NOCCD method with respect to the CCSD[RHF] approximation.

As seen in Figure 1, the reference weight is close to unity for distances up to about 2.5 Å. At greater distances, the reference weight drops, falling below 50% for both the CCSD[RHF] and CCSD[RKS] methods. With the CCSD[RHF] model, the weight is transferred roughly equally to *W*_1_ and *W*_2_, indicating significant approximate orbital relaxation due to . Indeed, the -transformed reference weight is substantially greater than the untransformed one. The same effect is observed with the CCSD[RKS] model, although much less pronounced. The singles weight increases but much less than in the CCSD[RHF] case. As one might perhaps have expected, the -transformed reference and doubles weights are roughly the same for the CCSD[RHF] and CCSD[RKS] models. With mean absolute deviations of 0.02 for *W*_0_ and 0.01 for *W*_2_, the NOCCD model predicts weights that closely agree with the -transformed weights of the CCSD[RHF] and CCSD[RKS] theories. Hence, the CCSD[RHF], CCSD[RKS], and NOCCD approximations provide the same qualitative picture of the correlated ground state of LiH across the potential-energy surface.We next turn to the C_2_ molecule where the CCSD approximation fails due to multireference character. The calculations are done in the same way as the LiH ones above, and with the same basis set (cc-pVTZ). Some deviations are seen already at the CCSD[RHF] equilibrium bond distance *R*_e_ = 1.242 Å where the CCSD[RKS] approach predicts an energy 2.42 mE_h_ (6.34 kJ/mol) above the CCSD[RHF] energy. The NOCCD energy is somewhat higher, at 2.53 mE_h_ (6.63 kJ/mol) above the CCSD[RHF] energy. At *R* = 2.732 Å, the CCSD[RKS] energy is 0.15 mE_h_ (0.39 kJ/mol) below the CCSD[RHF] one, while the NOCCD energy is 2.30 mE_h_ (6.03 kJ/mol) below.

**Figure 2:**
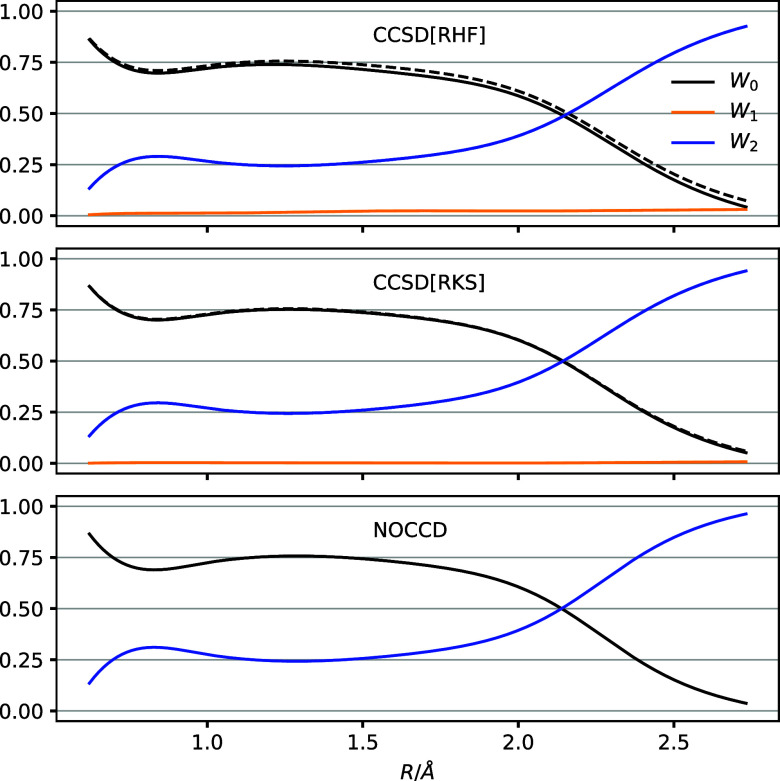
Same as Figure 1, here for the C_2_ molecule.

The weights are plotted in Figure 2. At *R*_e_, the CCSD[RHF] approximation predicts a reference weight of roughly 75%, with the remaining 25% residing mainly in double-excited determinants and very little in single-excited determinants. This picture is also obtained with the RKS reference, albeit with even less population in the single-excited determinants. Also the NOCCD method agrees. The singles weight increases a bit in the CCSD[RHF] state as the bond length is increased, whereas it remains negligible at all bond distances in the CCSD[RKS] state. Hence, the  operator can do only little to improve the reference. All three methods agree that the reference weight nearly vanishes at *R* = 2.732 Å, with the doubles weight reaching close to 100%. This, of course, indicates a strongly correlated system, although one must always keep in mind the orbital-dependence of the weights.

Regardless of the reference choice, at *R* = 2.732 Å, the CCSD doubles weight is dominated by four distinct combinations of π–π* (HOMO–LUMO) double excitations. One of them turns out to be negative, −0.113 with the RKS reference and −0.106 with the RHF reference. Such out-of-bounds weights can be taken as an indication that the state is poorly described with the CCSD approximation (keeping in mind the inherent orbital-dependence, of course). Along these lines, it should be noted that the total *W*_0_ and *W*_2_ would become negative and greater than 1, respectively, if the bond distance is further increased in Figure 2, for all three methods. This merely illustrates that one cannot remedy multireference character by choosing a single reference determinant.

Large doubles amplitudes τ_μ_2__ have long been taken as an indication of strong correlation or potential multireference character, although the precise and general definition of “large” remains unclear. It is interesting to note that the doubles amplitudes corresponding to the dominant π–π* doubles weights for C_2_ at *R* = 2.732 Å are also by far the largest amplitudes, accounting for more than 80% of the total (Frobenius) norm of the entire amplitude array. However, the amplitude corresponding to the determinant with the greatest weight is not the one with the greatest amplitude value. In fact, it only accounts for about 10% of the total amplitude norm, illustrating the difficulties faced when trying to define the precise meaning of “large” doubles amplitudes.

### 3.4 Noninteracting Subsystems

To elucidate the separability issues associated with the linear parametrization of , we consider the H_2_ dimer. The two hydrogen molecules both have bond distance *R* and are placed in a parallel configuration with a separation denoted *D*. That is, the four protons form a rectangle with side lengths *R* and *D*. Choosing D = 1000 a_0_, the two hydrogen molecules can be considered noninteracting.

By size-consistency, the CCSD energy of the dimer will be equal to twice the monomer energy. Since H_2_ is a two-electron system, the monomer energy will be equal to the exact result, the FCI energy. Hence, the CCSD energy of the dimer will be exact for all values of *R*. Due to the linear parametrization of , however, the bivariational CCSD ground state of the dimer is not exact, and separability issues are expected to arise in the weights. Since the H_2_ molecules are effectively noninteracting, the components missing in the linear Λ̂ operator are disconnected triples and quadruples. These are included in the QCCSD model, albeit in an approximate fashion. Hence, the QCCSD model should yield both the exact energy and a much-improved approximation of the weights.

For reference, Table 6 contains the FCI energies and weights obtained for the H_2_ molecule with the cc-pVDZ basis set.

**Table 6:** Reference, Singles, and Doubles Weights for the H_2_ Molecule Obtained from the FCI Wave Function with the cc-pVDZ Basis^a^

*R*/*R*_e_	*E*/*E*_h_	*W*_0_	*W*_1_	*W*_2_
1	–1.16339873	0.98311	0.00010	0.01678
2	–1.06392796	0.91291	0.00268	0.08441
4	–0.99966961	0.56362	0.01551	0.42086

a The equilibrium bond distance is *R*_e_ = 1.4 a_0_.

The energies and weights obtained from the CCSD (and QCCSD) method are identical and, therefore, not reported. Using eqs 17–19, we can easily predict the reference, singles, and doubles weights that should be obtained for the noninteracting dimer. Our results for the H_2_ dimer are reported in Table 7.

**Table 7:** Reference, Singles, Doubles, Triples, and Quadruples Weights for Two Noninteracting H_2_ Molecules (Separated by 1000 a_0_)^a^

	*R*/*R*_e_	CCSD	QCCSD	FCI
*W*_0_	1	0.96622	0.96651	0.96651
	2	0.82582	0.83340	0.83340
	4	0.12721	0.31773	0.31767
*W*_1_	1	0.00021	0.00021	0.00021
	2	0.00536	0.00489	0.00489
	4	0.03109	0.01737	0.01749
*W*_2_	1	0.03357	0.03300	0.03300
	2	0.16883	0.15413	0.15413
	4	0.84170	0.47465	0.47466
*W*_3_	1	0.00000	0.00000	0.00000
	2	0.00000	0.00045	0.00045
	4	0.00000	0.01316	0.01306
*W*_4_	1	0.00000	0.00028	0.00028
	2	0.00000	0.00713	0.00713
	4	0.00000	0.17708	0.17713

a The monomer equilibrium bond distance is *R*_e_ = 1.4 a_0_. All results are obtained with the cc-pVDZ basis set.

The energies obtained from the CCSD, QCCSD, and FCI methods are identical and equal to twice the monomer energies reported in Table 6, as required by size-consistency. It is easily verified that the FCI reference, singles, and doubles weights exactly satisfy eqs 17–19.

For the CCSD method, however, we observe deviations due the lack of multiplicative separability of . While the deviations are almost negligible at *R* = *R*_e_, they increase rapidly with *R*, and at *R* = 4*R*_e_, the reference, singles, and doubles weights are off by roughly a factor of 2.

The QCCSD method yields a significant improvement, almost exactly reproducing the FCI results for *W*_0_, *W*_1_, and *W*_2_ at all *R*. The greatest deviation is on the order of 10^–4^ for the reference and singles weights at *R* = 4*R*_e_. With the QCCSD method we can also compare the triples and quadruples weights with the FCI results. Also for these, we observe an excellent agreement.

As mentioned above, the CCSD method provides an excellent approximation to the FCI energy for the LiH molecule. If we consider the LiH dimer in a noninteracting rectangular configuration analogous to the one used for the H_2_ dimer above, the CCSD dimer energy remains accurate thanks to size-consistency. The LiH molecule, however, is a four-electron system and the CCSD Ansatz is not formally exact. The two core electrons only contribute very little to the electron correlation energy as the bond distance is increased and, consequently, the LiH molecule can be seen as almost a two-electron system in this context.

**Figure 3:**
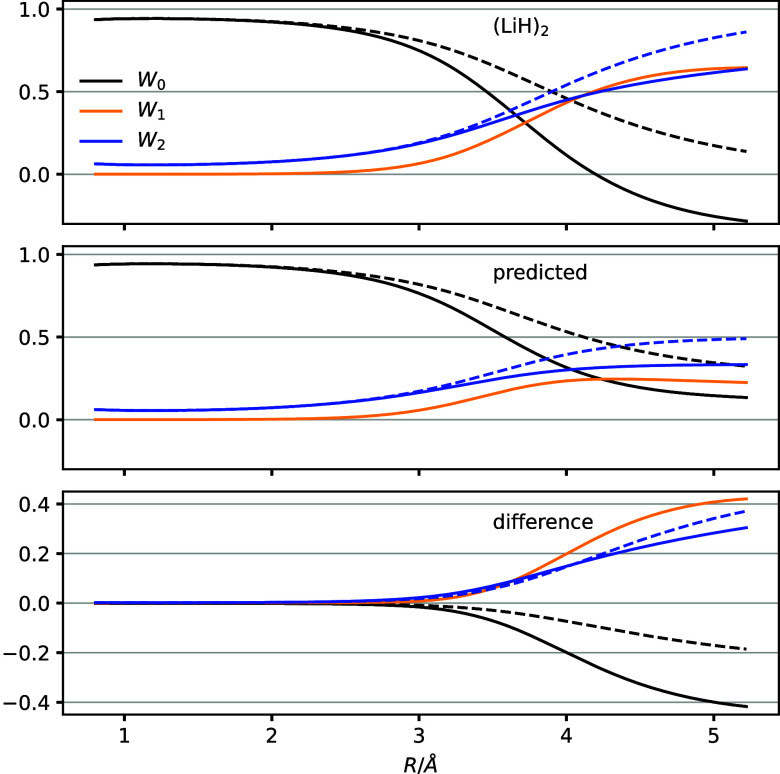
Weights computed for the noninteracting LiH dimer, presented as in Figure 1. The top panel shows results obtained with the CCSD method using the RHF reference and the cc-pVTZ basis set. The middle panel shows the results predicted by eqs 17–19 using the monomer data in the top panel of Figure 1. The last panel shows the difference between the two.

We present CCSD weights for the LiH dimer as functions of the Li–H distance *R* in Figure 3.The behavior of the weights as functions of *R* is qualitatively similar to the monomer case presented in Figure 1, but we immediately notice that the reference weight becomes negative at distances beyond roughly 4 Å. The -transformed weights remain within bounds, however, at least at the distances considered.

Using eqs 17–19 to predict the dimer weights clearly does not produce negative weights at large distances. Rather, the predicted reference, singles, and doubles weights appear to converge to values well within bounds at large *R*. The difference between the computed and predicted dimer weights are negligible or small for distances up to about twice the LiH equilibrium distance *R*_e_ = 1.5958 Å, however.

**Figure 4:**
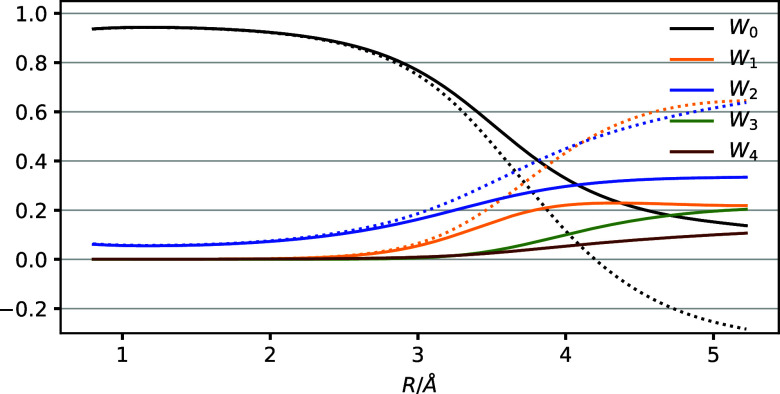
QCCSD reference, singles, doubles, triples, and quadruples weights computed for the noninteracting LiH dimer with the cc-pVTZ basis set. The dotted lines show the CCSD weights for comparison.

As can be seen in Figure 4, the weights obtained with the QCCSD method remain within bounds, in marked contrast to the conventional CCSD method. As above, this is due to the disconnected triples and quadruples de-excitations, which cause significant triples and quadruples weights at large Li–H distances with concomitant changes in the reference, singles, and doubles weights. The effective two-electron nature of the LiH monomer reveals itself through QCCSD reference, singles, and doubles weights that are almost identical to those predicted from the CCSD monomer data in Figure 1. The maximum relative deviation between the QCCSD and predicted CCSD weights are 6% for the reference, 7% for the singles, and 2% for the doubles.

## 4 Summary and Concluding Remarks

We have demonstrated that weights can be defined within CC theory as bivariational expectation values of projection operators. This allows for a wave function analysis analogous to configuration-interaction-based models for all approximate CC models, including those that are based on perturbation theory (e.g., the CCSD(T) method) and thus do not provide an explicitly parametrized right (ket) or left (bra) wave function. We note, however, that weights cannot be used as strict diagnostics for multireference character, as they are neither size-consistent nor invariant to the choice of orbital basis. The latter applies, of course, to both CC theory and configuration-interaction-based theories, including FCI theory. The orbital-dependence can be turned into an advantage, since the weights nicely capture the effect of the choice of orbital basis. In particular, the well-known orbital-relaxation effect of the  operator is easily seen to correct short-comings of the chosen reference determinant in such a way that nearly the same energy is obtained with any reasonable reference.

The main disadvantage of the CC weights concept is the lack of proper separability for noninteracting subsystems, a concept closely related to size-consistency. The culprit is the linear parametrization of the left (bra) state, , which breaks the multiplicative separability observed for the FCI wave function. Only at the untruncated (full CC) limit is separability guaranteed. Most likely, the only way to correct this behavior is to use Arponen’s extended CC theory. This is corroborated by results obtained with quadratic CC theory where we observe a much-improved behavior.

One might perhaps argue that the lack of proper separability tells one that expectation values of operators that are not additively separable—such as the projection operators defining the CC weights—should not be computed in the usual CC manner, i.e., using the bivariational form, eq 7, with the linear Λ̂ operator. However, the linear operator naturally appears for both ground and excited states in the widely used EOM-CC theory,^18−20^ despite the associated separability issues.^80,81^ In addition, it should be recalled that the CC one-electron density matrix consists of elements defined as expectation values
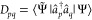
72of products of creation and annihilation operators, which are neither additively nor multiplicatively separable. The eigenvalues of this matrix (often after symmetrization as dictated by eq 7) are interpreted as natural occupation numbers and used to, e.g., define indices of multideterminant and multireference character.^36^ As an example, the natural occupation numbers for the LiH dimer discussed above should be exactly identical to those obtained for the monomer (repeated twice, of course). The norm of the vector measuring the difference between computed and expected (from monomer calculations) natural occupation numbers for the LiH dimer increases by 2 orders of magnitude from *R* = *R*_e_ to *R* = 3.25*R*_e_. (The deviations are small enough to be negligible in this case, though: 2.3 × 10^–9^ at *R* = *R*_e_ and 1.6 × 10^–7^ at *R* = 3.25*R*_e_.)

We conclude that the weight concept appears as a useful tool for analyzing CC states, although one needs to be aware of the separability issues and orbital-dependence (which is always an issue, also for configuration-interaction methods). In practice, our tests indicate that a CC calculation may be assumed to be reliable if the reference weight is close enough to unity, especially if the reference weight is computed in the -transformed basis. Reference weights further from unity may indicate multireference character and potential failure of the single-reference CC method. However, it may also be a consequence of fundamental separability issues that do not necessarily imply poor energies. Further testing, particularly for larger systems, is clearly needed to establish the usability of the reference weight in this regard.

The weight concept can be straightforwardly extended to EOM-CC theory,^18−20^ thus providing a simple and systematic characterization of excited states in terms of electron configurations. Currently, this is usually done by judging the relative magnitudes of the components of the EOM-CC eigenvectors. For the same reason, the weight concept can be used to interpret CC simulations—using either TDCC or time-dependent EOM-CC theory^8^—of many-electron dynamics in terms of elementary orbital transitions, which is the language most commonly used in experimental chemistry. This includes assignment of absorption lines obtained from the Fourier transform of the induced dipole moment.

Finally, we note that CC weights may be useful for analyzing electron-correlation effects in ground and excited states using quantities such as the Shannon entropy from quantum information theory.^82^

## Appendix A

### Algebraic CCSDT Expressions in Spin–Orbital Basis

In the CCSDT approximation

A1

where, with indices *a*, *b*, *c* denoting virtual spin orbitals and *i*, *j*, *k* occupied ones

A2

A3

Then, computing *c*_μ_ = ⟨Φ_μ_|Ψ⟩ and  for μ ∈ {0, 1, 2, 3}, we obtain

A4

A5

A6

A7

and

A8

A9

A10

A11

The CCSD weights are easily obtained from these expressions by putting the triples amplitudes equal to zero.
